# Geographic variation of inpatient care costs at the end of life

**DOI:** 10.1093/ageing/afw040

**Published:** 2016-03-28

**Authors:** Claudia Geue, Olivia Wu, Alastair Leyland, Jim Lewsey, Terry J. Quinn

**Affiliations:** 1 Health Economics and Health Technology Assessment, University of Glasgow, Glasgow G12 8RZ, United Kingdom of Great Britain and Northern Ireland; 2 MRC/CSO Social and Public Health Sciences Unit, University of Glasgow, Glasgow, United Kingdom of Great Britain and Northern Ireland; 3 Institute of Cardiovascular and Medical Sciences, University of Glasgow, Glasgow, United Kingdom of Great Britain and Northern Ireland

**Keywords:** healthcare expenditure, end of life, geography, older people

## Abstract

**Background:** costs incurred at the end of life are a main contributor to healthcare expenditure. Urban–rural inequalities in health outcomes have been demonstrated. Issues around geographical patterning of the association between time-to-death and expenditure remain under-researched. It is unknown whether differences in outcomes translate into differences in costs at the end of life.

**Methods:** we used a large representative sample of the Scottish population obtained from death records linked to acute inpatient care episodes. We performed retrospective analyses of costs and recorded the most frequent reasons for the last hospital admission. Using a two-part model, we estimated the probability of healthcare utilisation and costs for those patients who incurred positive costs.

**Results:** effects of geography on costs were similar across diagnoses. We did not observe a clear gradient for costs, which were lower in other urban areas compared with large urban areas. Patients from remote and very remote areas incurred higher costs than patients from large, urban areas. The main driver of increased costs was increased length of stay.

**Conclusions:** our results provide evidence of additional costs associated with remote locations. If length of stay and costs are to be reduced, alternative care provision is required in rural areas. Lower costs in other urban areas compared with large urban areas may be due to urban centres incurring higher costs through case-mix and clinical practice. If inequalities are driven by hospital admission, for an end of life scenario, care delivered closer to home or home-based care seems intuitively attractive and potentially cost-saving.

## Introduction

Previous research has shown that costs incurred at the end of life, in particular for inpatient care, are a main contributor to total healthcare expenditure (HCE) [1–8]. Increasing HCE in the context of population ageing is not solely driven by advanced age, rather older adults tend to be closer to death [9]. Understanding patterns and contributors to end of life and associated HCE has important implications for clinicians, local services and national policy.

Ensuring appropriate and equitable care is provided across all geographic areas within a country is important but can be difficult to achieve in practice. Several studies have described differences in mortality dependent on where people lived [10–13]. Rural to urban differences in morbidity and mortality may be partly explained by healthcare utilisation. For example, there is evidence of a direct association between the distance and travel time to hospitals, and increased mortality from acute myocardial infarction (AMI) [10, 11] and stroke [12] so that geographical disparity and related distance from hospital and other aspects of access to HC services seem plausible.

There is additional evidence on local variation in spending on HC services for older people with higher spending per capita reported in Orkney and Shetland compared with all other mainland areas [14]. What is less known is where differences in outcomes and overall spending stem from and whether end of life care in acute or inpatient care settings are the main contributor. Two scenarios seem possible: (i) increased costs for inpatient care due to remoteness and related challenges (e.g. difficulties in discharge planning due to increased length of stay (LOS)), or (ii) reduced costs in the context of increased early mortality (e.g. patients not hospitalised in time to receive emergency care) or differences in the delivery of care (e.g. it seems plausible that for the same diagnosis, patients from rural areas are admitted to hospital less frequently than patients from urban areas). Both situations are important, but the healthcare and policy implications would be very different. In particular in rural settings, this might support alternative pathways of care, such as community-based alternatives instead of hospital care [14]. To date, the geographical patterning of the association between remaining time to death (TTD), age and HCE has not been well described.

Scotland is well suited to descriptive work looking at the association between health service utilisation and geographic location, with ∼21% of the population living in rural or remote areas. It has been shown that geographical variation in mortality is higher in Scotland than in the rest of the UK [13]. Health inequalities among people aged 65 and older are greater in remote rural Scotland than in urban parts of the country [15]. A specific focus on inpatient care costs can be useful as the majority of costs in patients discharged from acute medical units (predominantly frail elderly patients) were incurred in hospital (∼75% of overall costs) [16].

The aim of this study was to investigate whether geographic inequalities in mortality also translate into differences in costs incurred for the last admission to hospital prior to death.

## Methods

We used a longitudinal dataset comprising a 5% sample of all Scottish decedents derived from national mortality data (National Records Scotland; NRS) and randomly drawn by the Information and Services Division (ISD) Scotland. ISD linked these mortality data to inpatient hospital records (Scottish Morbidity Records; SMR01). Data on death, preceding hospital admissions and associated costs were available from 1997 to 2012. The costing method that we used employed Healthcare Resource Groups (HRGs) as the basis on which we assigned unit costs to a Continuous Inpatient Stay (CIS) [17]. These are standard groups of clinically similar diseases and treatments that use a common level of HC resources, which are widely used, and also known as Diagnosis Related Groups (DRGs) internationally. We assigned extra per diem costs where the stay exceeded the mean LOS for that particular HRG [16].

We identified the 10 most frequent reasons for the last admission to hospital prior to death for various geographical areas. The clinical data of interest were not the recorded cause of death, but the reason for admission to hospital as these better defines the costs incurred for that episode. We used the primary diagnostic code (categorised using International Classification of Disease 10 [ICD-10]) to identify the reasons for admission. These codes refer to the main medical condition that was managed during the hospitalisation [18]. To best capture disease classifications and to minimise errors due to recording variations, we used higher level classifications (three digit ICD-10 codes). If several episodes formed a CIS, the first episode was used to determine the primary reason for admission, and costs were estimated for the entire stay. We described differing geographical areas of interest using the 8-fold urban–rural classification for Scotland [19]. In this classification areas are defined as:

**Table:** 

Large urban area	Settlement with 125,000 people or more
Other urban area	Settlement with 10,000 to 124,999 people
Accessible small towns	Settlement with 3,000 to 9,999 people and within a 30 min drive to a settlement of >10,000 people
Remote small towns	Settlement with 3,000 to 9,999 and over 30 min drive to settlement of >10,000 people
Very remote small towns	Settlement with 3,000 to 9,999 people and over 60 min drive to settlement of >10,000 people
Accessible rural areas	Settlement of <3,000 people and within 30 min drive of settlement of >10,000 people
Remote rural areas	Settlement of <3,000 people >30 min drive to settlement of >10,000 people
Very remote rural areas	Settlement of <3,000 people and >60 min drive to settlement of >10,000 people

Although this classification does not directly measure driving time or distance to the nearest hospital, it gives a very good estimation of driving time to the nearest bigger settlement, and this can be used as a proxy for distance to major hospital site and overall accessibility of HC services. For our analysis, we ordered the 8-fold urban–rural categorisation in terms of driving time to the next larger settlements.

We based our analyses on hospital admissions over the last 3 years of life, identifying the last admission prior to death within this time period. We measured TTD in quarters, with each individual patient contributing at least 12 observations. If there was no hospitalisation in a particular quarter, these were assigned zero costs. To obtain unbiased estimates of costs, these observations were included in the analysis and a two-part model was employed, estimating the probability of hospital utilisation in its first part (using a probit link and a binomial distribution) conditional on a set of explanatory variables X (Equation 1). Costs were then estimated in the second part conditional on positive utilisation.

(1) $$\mathrm{Pr}(\text{HC}{{\text{E}}_{i}}_{,t}>0)=\Phi \left(\begin{array}{cc} & \alpha_{0}+\sum_{a=2}^{8}\alpha_{1}A_{it}+\alpha_{2}S_{i}+\alpha_{3}\text{TT}{\text{D}}_{i}\\ & +\sum_{u=2}^{8}\alpha_{4}U_{it}+\sum_{d=2}^{10}\alpha_{5}D_{i}+\alpha_{6}Y_{i}+u_{i} \end{array}\right)$$

where *A* = age at death categories (reference category: <45 years); S = sex (reference category: male); TTD = remaining time to death in quarters (reference category: 12th quarter before death); U = urban/rural indicator (reference category: large urban area); *D* = Scottish Index of Multiple Deprivation (SIMD) deciles (reference category: most deprived decile (1)); *Y* = time period of admission; *u_i_* = robust standard errors for patient *i* at time *t*_._

From the second part of the model, costs were estimated for those patients who incurred positive costs; i.e. greater than zero and expected costs were obtained for each decedent. The same explanatory variables were used in the second modelling part (Equation 2).

(2) E[HCE]=g(xβ)

with *xβ* representing the linear predictor for HCE. We estimated costs by fitting a generalised linear model (GLM) clustered on patient identifier with a Gamma distribution and a log link. The distributional family takes into account the heavily skewed nature of HCE data and clustering accounts for multiple hospital admissions for the same patient. In a third step, both modelling parts were multiplied, and cost estimates were recorded for each of the geographical regions (Equation 3). Separate models were estimated for each disease classification.

(3) E[HCE|X]=Pr(HCE>0|X)∗E(HCE|HCE>0,X)

We calculated the total cost per disease by multiplying individual-level estimates for each area with their respective sample sizes and aggregated over areas. We then calculated the proportional share of costs for each area.

All analyses were undertaken using Stata/SE12.1 software (STATA Corp, TX, USA). A *P* value of <0.05 was considered to indicate a statistically significant difference.

## Results

Hospital admission records and related costs (1997–2012) were available for 39,366 patients with a total number of related observations of 618,333 over the last 3 years of life (Supplementary data, Table S1, available in *Age and Ageing* online). The group not accessing hospital prior to death (*n* = 1,986) were more likely to be younger, male and lived in rural areas.

**Figure 1.**
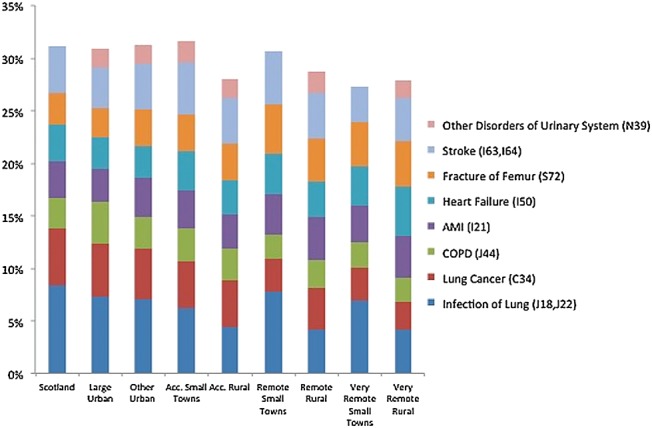
Most frequent reason (ICD10, 3 digits) for last admission to hospital prior to death by urban–rural classification and for Scotland (overall).

The most frequent diagnoses for the last admission to hospital prior to death are shown in Figure 1. Diagnostic codes for cerebral infarction (I63) and stroke (I64) were combined, and we report results for ‘stroke’. We also combined ICD codes for pneumonia (J18) and lower respiratory infection (J22) into ‘infection of lung’. The most frequent admission reasons were infection of the lung: stroke and lung cancer. Overall, reasons of admission did not seem to vary markedly between urban and rural areas (Figure 1).

After adjusting for age, sex, year of admission, social deprivation (Scottish Index of Multiple Deprivation SIMD) and remaining TTD, the overall effects of urban–rural area classification on costs for the last admission to hospital were similar across diagnoses (Supplementary data, Table S2, available in *Age and Ageing* online). Costs are shown as estimated costs from the two-part model with associated *P* values, sample size and proportional share of overall costs.

**Figure 2.**
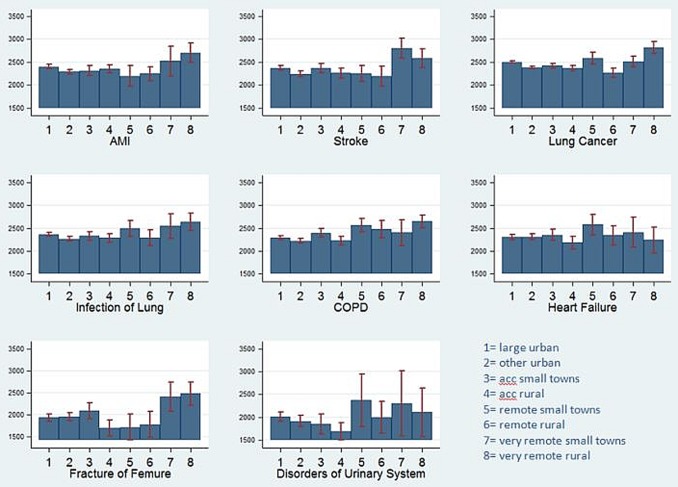
Estimated costs (GBP) for last admission to hospital prior to death during a 3-year observational period by geographical area type (95% CI indicated through error bars).

Costs in very remote, rural areas were significantly higher than costs in large, urban areas (Figure 2). We found costs for AMI, stroke, infection of lung and fracture of femur to be highest in very remote, rural areas and very remote small towns, whereas costs for lung cancer, chronic obstructive pulmonary disease (COPD), heart failure and other disorders of the urinary system also showed an increase if patients lived in remote small towns. In addition, costs in other urban areas were lower than those in large urban areas for most diagnoses (*P* < 0.001). The proportional share of total costs is broadly similar across diseases, and large urban areas contribute most to overall costs.

A post hoc investigation of the main driver of increased costs in more remote areas revealed that LOS in hospital was longer in more remote, rural areas than in urban areas (Supplementary data, Figure S3, available in *Age and Ageing* online). We found that particularly for stroke, infections of the lung and disorders of the urinary system, LOS was markedly longer in very remote areas than in more accessible, urban areas.

## Discussion

We described differences in end-of-life HCE between geographical regions based on rurality, using rurality as an indicator for organisation of care and distance. Our results suggest that for the common medical conditions that precipitate hospital admission at the end of life, rurality had a significant effect on estimated costs. We also showed that these medical conditions are in line with causes of admission recorded for the whole of Scotland.

Our data provide further evidence of urban–rural differences in healthcare utilisation and outcomes. This occurs in a context of increasing moves towards specialisation and centralisation of clinical services. We did not observe a clear gradient for costs, rather, costs seemed to be lower in other urban areas compared with large urban areas, possibly due to large urban centres having high costs because of case-mix, clinical practice and the fact that patients might be more readily admitted to hospital if living in large urban areas.

Our study was not specifically designed to look at reasons for these differences, and further work around the mechanisms that drive differing HCE will be required to inform healthcare policy. However, we can offer some plausible reasons. The urban–rural categorisation we employed operationalises levels based on distance to large settlement and so we infer that increasing rurality equates to increasing distance from hospital. A post hoc investigation of the main driver of these differences revealed that LOS in hospital was longer in very remote rural areas, which is in line with previous research that found that patients in rural areas experienced longer hospital stays on average [15]. It also supports our initial hypothesis of increased LOS due to remoteness and related challenges. Our study was not designed to suggest solutions to these potential geographical inequalities. However, if inequalities are driven by the hospital admission, then for an end-of-life scenario care delivered closer to home would seem intuitively attractive. Unsurprisingly, proportional shares of total costs were highest for urban areas due to population numbers; however, since the healthcare resource allocation formula already adjusts for population size, any savings that could be made through alternative care arrangement are expected to have a substantial impact on costs. Research from England has shown that access to non-acute care at the end of life, such as home-based end of life care can reduce hospital admissions and save costs [20]. A recent study from Scotland accentuates this issue and found that at any one time ∼30% of patients in hospital are in the last year of their life and highlights the importance of developing appropriate care plans [21]. This study also raised the issue of the right place and time to identify these patients.

The strengths of our analyses were our use of a large and representative sample of the Scottish population with robust data on healthcare utilisation. Our approach has limitations; using Scotland as the basis for our analysis meant that we had a varied urban–rural case-mix and could benefit from high-quality national administrative data collection. However, this also means that our findings may not be immediately generalisable to other settings, particularly where access to health care is dependent on insurance or personal financial resource. For example, the rural population in Scotland tends to be more affluent than the urban population; this is in contrast to Australia, another country that faces particular challenges delivering care to remote and rural locations [22]. In addition, we acknowledge that the relationship between geographic area and costs is multifaceted and cannot solely be explained by distance from hospital. We have controlled for a number of additional factors; however, we were not able to account for factors related to the organisation and delivery of health care in different areas. Furthermore, we appreciate that only estimating costs for inpatient care neglect the social care component that is important for end-of-life care. However, we do feel that our findings of high costs at the end of life and variations of costs by geographic area emphasise the importance of alternative pathways of care towards the end of life. By focusing on the last admission before death, we might underestimate the impact that rurality has on costs; however, since dying in hospital is one of the main cost drivers, we believe this to be the most important period in terms of cost differences. It also reinforces the argument for alternative community-based care options in particular in rural areas.

Overall, we can conclude that an increased risk of mortality for patients being admitted to hospital from rural areas, as reported in the literature, does not seem to lead to reduced costs due to non-admission. On the contrary, once admitted to hospital, patients living in remote areas incurred higher costs than those from large urban areas. This highlights the difficulties that rural areas face when discharging patients at the end of life in areas where there might be a lack of end-of-life support facilities as these are more frequently available in larger urban areas.

**Key points**

- A substantial proportion of HCE is incurred at end of life.
- Geographical differences in healthcare costs are apparent, with increasing rurality associated with higher costs at end of life.
- Rural areas face challenges in providing end-of-life care in alternative settings, leading to increased hospital costs.

## Supplementary data

Supplementary data mentioned in the text are available to subscribers in *Age and Ageing* online.

## Conflicts of interest

None declared.

## Funding

This work was supported through a post-doctoral fellowship awarded by the Chief Scientist Office of the Scottish Government Health and Social Care Directorate (PDF/13/03). A.L. is funded by the Medical Research Council and the Chief Scientist Office of the Scottish Government Health and Social Care Directorate (MC_UU_12017/13).

## Supplementary Material

Supplementary Data

## References

[1] Payne G, Laporte A, Deber R, Coyte PC Counting backward to health care's future: using time-to-death modelling to identify changes in end-of-life morbidity and the impact of aging on health care expenditures. Milbank Q 2007; 85: 213–57. 17517114 10.1111/j.1468-0009.2007.00485.x PMC2690327

[2] Zweifel P, Felder S, Meiers M Ageing of population and health care expenditure: a red herring? Health Econ 1999; 8: 485–96. 10544314 10.1002/(sici)1099-1050(199909)8:6<485::aid-hec461>3.0.co;2-4

[3] Zweifel P, Felder S, Werblow A Population ageing and health care expenditure: new evidence on the ‘Red Herring’. Geneva Papers Risk Insur 2004; 29: 652–66.

[4] Seshamani M, Gray AM Ageing and health-care expenditure: the red herring argument revisited. Health Econ 2004; 13: 303–14. 15067669 10.1002/hec.826

[5] Seshamani M, Gray A Time to death and health expenditure: an improved model for the impact of demographic change on health care costs. Age Ageing 2004; 33: 556–61. 15308460 10.1093/ageing/afh187

[6] Breyer F, Lorenz N, Niebel T Health Care Expenditures and Longevity: Is There a Eubie Blake Effect? Discussion Paper 1226, 2012 DIW Berlin. 10.1007/s10198-014-0564-x 24585039

[7] Moorin RE, Holman CD The cost of in-patient care in Western Australia in the last years of life: a population-based data linkage study. Health Policy 2008; 85: 380–90. 17913279 10.1016/j.healthpol.2007.08.003

[8] Moorin R, Gibson D, Holman D, Hendrie D The contribution of age and time-to-death on health care expenditure for out-of-hospital services. J Health Serv Res Policy 2012; 17: 197–205. 23038709 10.1258/jhsrp.2012.011130

[9] Geue C, Briggs A, Lewsey J, Lorgelly P Population ageing and healthcare expenditure projections: new evidence from a time to death approach. Eur J Health Econ 2014; 15: 885–96. 24292437 10.1007/s10198-013-0543-7

[10] Nicholl J, West J, Goodacre S, Turner J The relationship between distance to hospital and patient mortality in emergencies: an observational study. Emerg Med J 2007; 24: 665–8. 17711952 10.1136/emj.2007.047654 PMC2464671

[11] Wei L, Lang CC, Sullivan FM et al Impact on mortality following first acute myocardial infarction of distance between home and hospital: cohort study. Heart 2008; 94: 1141–6. 17984217 10.1136/hrt.2007.123612 PMC2564842

[12] Votruba ME, Cebull RD Redirecting patients to improve stroke outcomes: implications of a volume-based approach in one urban market. Med Care 2006; 44: 1129–36. 17122718 10.1097/01.mlr.0000237424.15716.47

[13] Leyland AH Increasing inequalities in premature mortality in Great Britain. J Epidemiol Commun Health 2004; 58: 296–302. 10.1136/jech.2003.007278 PMC1732731 15026442

[14] Reshaping Care for Older People. 2014 Auditor General Audit Scotland http://www.audit-scotland.gov.uk/docs/central/2014/nr_140206_reshaping_care.pdf (29 February2016, date last accessed).

[15] Levin KA, Leyland AH A comparison of health inequalities in urban and rural areas in Scotland. Soc Sci Med 2006; 62: 1457–64. 16182421 10.1016/j.socscimed.2005.08.045

[16] Franklin M, Berdunov V, Edmans J et al Identifying patient-level health and social care costs for older adults discharged from acute medical units in England. Age Ageing 2014; 43: 703–7. 25059421 10.1093/ageing/afu073

[17] Geue C, Lewsey J, Lorgelly P, Govan L, Hart C, Briggs A Spoilt for choice: implications of using alternative methods of costing hospital episode statistics. Health Econ 2012; 21: 1201–16. 21905152 10.1002/hec.1785

[18] Information Services Division. SMR Datasets. General Clinical Information. http://www.ndc.scot.nhs.uk/Data-Dictionary/SMR-Datasets/General-Clinical-Information/Diagnostic-Section/Main-Condition.asp (5 March 2015, date last accessed).

[19] Information Services Division. GPD Support. Geography. Urban-Rural Classification. http://www.isdscotland.org/Products-and-Services/GPD-Support/Geography/Urban-Rural-Classification/ (5 March 2015, date last accessed).

[20] Chitnis XA, Georghiou T, Steventon A, Bardsley MJ Effect of a home-based end-of- life nursing service on hospital use at the end of life and place of death: a study using administrative data and matched controls. BMJ Support Palliat Care 2013; 3: 422–30. 10.1136/bmjspcare-2012-000424 24950522

[21] Clark D, Armstrong M, Allan A, Graham F, Carnon A, Isles C Imminence of death among hospital inpatients: prevalent cohort study. Palliat Med 2014; 28: 474–9. 24637342 10.1177/0269216314526443 PMC4845030

[22] Wakerman J, Humphreys JS Sustainable primary health care services in rural and remote areas: Innovation and evidence*.* Aust J Rural Health 2011; 19: 118–24. 21605224 10.1111/j.1440-1584.2010.01180.x

