# Supplementary material for: Geographic variation of inpatient care costs at the end of life
Source: Age Ageing. 2016 Mar 28;45(3):376–81. doi: 10.1093/ageing/afw040 (PMC4846794; doi:10.1093/ageing/afw040)
Supplement: Supplementary Data [file supp_45_3_376__index.html]

Geographic variation of inpatient care costs at the end of life — Geographic variation of inpatient care costs at the end of life — Supplementary Data 

# Geographic variation of inpatient care costs at the end of life

## Supplementary Data

Supplementary Data

- Supplementary Data - Docx file
