# Supplementary material for: Geographic variation of inpatient care costs at the end of life
Source: Age Ageing. 2016 Mar 28;45(3):376–81. doi: 10.1093/ageing/afw040 (PMC4846794; doi:10.1093/ageing/afw040)
Supplement: Supplementary Data [file supp_afw040_afw040supp.docx]

**Supplementary Data Table 1: Descriptive Statistics for hospital service users and non-users**

|  | **Hospitalised** | **Not Hospitalised** |
| --- | --- | --- |
|  | N (%) | N (%) |
|  | 37,380 (94.96) | 1,986 (5.04) |
| **Age at Death (years)** |  |  |
| <45 | 1,381 (3.69) | 441 (22.21) |
| 45 to 64 | 5,543 (14.83) | 406 (20.44) |
| 65 to 69 | 3,322 (8.89) | 180 (9.06) |
| 70 to 74 | 4,682 (12.53) | 221 (11.13) |
| 75 to 79 | 5,954 (15.93) | 200 (10.07) |
| 80 to 84 | 6,437 (17.22) | 205 (10.32) |
| 85 to 89 | 5,529 (14.79) | 173 (8.71) |
| >90 | 4,532 (12.12) | 160 (8.06) |
| **Sex** |  |  |
| Male | 17,716 (47.39) | 1,106 (55.69) |
| Female | 19,664 (52.61) | 880 (44.31) |
| **Urban-Rural Indicator** |  |  |
| Large urban ^(1)^ | 15,102 (40.40) | 781 (39.33) |
| Other urban ^(2)^ | 11,326 (30.30) | 533 (26.84) |
| Acc small town ^(3)^ | 3,226 (8.63) | 176 (8.86) |
| Accessible rural ^(4)^ | 3,701 (9.90) | 233 (11.73) |
| Remote small town ^(5)^ | 1,130 (3.02) | 52 (2.62) |
| Remote rural ^(6)^ | 1,173 (3.14) | 96 (4.83) |
| Very remote small town ^(7)^ | 519 (1.39) | 34 (1.71) |
| Very remote rural ^(8)^ | 1,176 (3.15) | 79 (3.98) |
| Missing | 27 (0.07) | 2 (0.10) |
| **SIMD Decile** |  |  |
| 1  2 | 5,240 (14.02) | 314 (15.81) |
| 2 | 4,696 (12.56) | 228 (11.48) |
| 3 | 4,504 (12.05) | 208 (10.47) |
| 4 | 4,320 (11.56) | 219 (11.03) |
| 5 | 3,837 (10.26) | 168 (8.46) |
| 6 | 3,456 (9.25) | 195 (9.82) |
| 7 | 3,165 (8.47) | 197 (9.92) |
| 8 | 2,970 (7.95) | 169 (8.51) |
| 9 | 2,604 (6.97) | 158 (7.96) |
| 10 | 2,561 (6.85) | 128 (6.45) |
| Missing | 27 (0.07) | 2 (0.10) |

**Supplementary Data Table 2: Estimated costs for last admission prior to death (p-values of cost ratios from two-part model)**

|  | **AMI (I21)** | | **Stroke (I63, I64)** | | **Lung Cancer (C34)** | | **Infection of Lung (J18, J22)** | | **Heart Failure (I50)** | | **COPD (J44)** | | **Fracture of Femur (S72)** | | **Other Disorders of Urinary System (N39)** | |
| --- | --- | --- | --- | --- | --- | --- | --- | --- | --- | --- | --- | --- | --- | --- | --- | --- |
|  | **£**  **(N)** | **p-value**  (% Total Costs) | **£**  **(N)** | **p-value**  (% Total Costs) | **£**  **(N)** | **p-value**  (% Total Costs) | **£**  **(N)** | **p-value**  (% Total Costs) | **£**  **(N)** | **p-value**  (% Total Costs) | **£**  **(N)** | **p-value**  (% Total Costs) | **£**  **(N)** | **p-value**  (% Total Costs) | **£**  **(N)** | **p- value**  (% Total Costs) |
| **Large urban  ^*^** | 2,403  (936) | n/a  (39.0) | 2,371  (1,292) | n/a  (40.6) | 2,503  (1,576) | n/a  (46.6) | 2,364  (2,449) | n/a  (44.0) | 2,311  (1,022) | n/a  (40.8) | 2,292  (911) | n/a  (46.3) | 1,939  (825) | n/a  (38.2) | 2,005  (463) | n/a  (44.1) |
| **Other urban** | 2,288  (759) | <0.001  (30.1) | 2,242  (927) | <0.001  (27.5) | 2,379  (1,032) | <0.001  (29.0) | 2,269  (1,720) | <0.001  (29.6) | 2,313  (739) | <0.001  (29.5) | 2,225  (594) | <0.001  (29.3) | 1,956  (637) | <0.001  (29.7) | 1,913  (278) | <0.001  (25.3) |
| **Accessible small towns** | 2,314  (210) | 0.723  (8.4) | 2,364  (304) | 0.856  (9.5) | 2,418  (281) | 0.725  (8.0) | 2,328  (451) | 0.770  (8.0) | 2,356  (232) | 0.746  (9.4) | 2,399  (154) | 0.881  (8.2) | 2,095  (192) | 0.701  (9.6) | 1,847  (113) | 0.710  (9.9) |
| **Accessible rural** | 2,350  (253) | 0.224  (10.3) | 2,262  (301) | 0.044  (9.0) | 2,367  (295) | 0.070  (8.2) | 2,288  (466) | 0.021  (8.1) | 2,182  (220) | 0.054  (8.3) | 2,227  (159) | 0.040  (7.8) | 1,707  (174) | 0.050  (7.1) | 1,688  (128) | 0.052  (10.3) |
| **Remote small towns** | 2,198  (92) | 0.062  (3.5) | 2,245  (132) | 0.040  (3.9) | 2,589  (86) | 0.053  (2.6) | 2,490  (170) | 0.080  (3.2) | 2,576  (79) | 0.054  (3.5) | 2,563  (44) | 0.051  (2.5) | 1,721  (78) | 0.065  (3.2) | 2,370  (17) | 0.066  (1.9) |
| **Remote rural** | 2,243  (89) | 0.519  (3.5) | 2,186  (115) | 0.969  (3.3) | 2,262  (89) | 0.984  (2.4) | 2,292  (169) | 0.995  (2.9) | 2,345  (87) | 0.788  (3.5) | 2,481  (45) | 0.988  (2.5) | 1,781  (73) | 0.990  (3.1) | 1,996  (42) | 0.991  (4.0) |
| **Very remote small towns** | 2,520  (30) | 0.120  (1.3) | 2,804  (46) | 0.081  (1.7) | 2,516  (33) | 0.126  (1.0) | 2,551  (65) | 0.105  (1.3) | 2,413  (40) | 0.106  (1.7) | 2,402  (22) | 0.072  (1.2) | 2,409  (59) | 0.083  (3.4) | 2,301  (12) | 0.112  (1.3) |
| **Very remote rural** | 2,706  (83) | <0.001  (3.9) | 2,585  (130) | <0.001  (4.4) | 2,816  (67) | <0.001  (2.2) | 2,637  (147) | <0.001  (2.9) | 2,244  (84) | <0.001  (3.3) | 2,648  (38) | <0.001  (2.2) | 2,479  (97) | <0.001  (5.7) | 2,105  (33) | <0.001  (3.3) |

*Reference Category

**Supplementary Data Figure 3: Length of stay (days) for last admission prior to death**
